# Exosomes derived from miR-1228 overexpressing bone marrow-mesenchymal stem cells promote growth of gastric cancer cells

**DOI:** 10.18632/aging.202878

**Published:** 2021-04-21

**Authors:** Lili Chang, He Gao, Li Wang, Ning Wang, Shumei Zhang, Xiaona Zhou, Huijun Yang

**Affiliations:** 1 Department of Gastroenterology, The First Ward, Shijiazhuang First Hospital, Shijiazhuang, Hebei Province, China; 2 Department of General Practice, Shijiazhuang First Hospital, Shijiazhuang, Hebei Province, China; 3 Department of Infectious Disease, Shijiazhuang First Hospital, Shijiazhuang, Hebei Province, China

**Keywords:** exosomes, bone marrow-mesenchymal stem cells, miR-1228, gastric cancer, MMP-14

## Abstract

There has been increasing evidence that microRNAs (miRNAs) are related to glioma progression, and that genetically engineered mesenchymal stem cells (MSCs) can inhibit the growth of gliomas. However, the underlying mechanism of bone marrow-MSCs (BM--MSCs) and miRs in gastric cancer still remains unclear. Patients with gastric cancer treated in Shijiazhuang First Hospital as well as healthy individuals undergoing physical examinations were recruited to measure the expression of exosomal miR-1228. Receiver operating characteristic (ROC) curves were plotted and the patients were followed up. BM--MSCs from healthy subjects were collected and exosomes were extracted. The MSC cells were transfected with lentiviral vectors carrying miR-1228 and MMP-14 over-expression sequences and scramble sequence, followed by exosome extraction. The exosomes were co-cultured with SGC-7901 and MGC-823 cells to detect cell proliferation, invasion, apoptosis and migration. The correlation between miR-1228 and MMP-14 was determined by dual-luciferase reporter assay. miR-1228 was highly expressed in serum exosomes of patients with gastric cancer with a area under ROC curve (AUC) of 0.865. The exosomes derived from BM-MSCs are expected to be efficient nanocarriers. Up-regulation of miR-1228 can down-regulate the expression of MMP-14 and effectively hinders the development and progression of gastric cancer.

## INTRODUCTION

With changes in people's living and eating habits, the prevalence of digestive system diseases has been rising. Gastric cancer, a common digestive system tumor, is one of the main causes of cancer-related deaths worldwide [1, 2]. There were more than 1 million new cases of gastric cancer and 780,000 deaths worldwide in 2018. Moreover, gastric cancer frequently presents at an advanced stage with malignant hyperplasia, which portends a poor prognosis [3, 4]. Therefore, it is urgent for clinicians to seek effective solutions.

MicroRNAs (miRs) are a class of highly conserved short non-coding RNAs with a length of approximately 17-25nt [5, 6], which affect the stability or inhibit the translation of mRNAs by regulating 3′ untranslated region (UTR) of their downstream target genes for complete or incomplete binding and complementation, thereby regulating the expression of proteins [7, 8]. Therefore, miRs have become a hot topic in various disciplines, especially in oncology. A study indicates that miR-21 is a potential tumor marker for gastric cancer [9], and another reveals that miR-101-2, miR-125b-2 and miR-451a are potential suppressors for gastric cancer via regulating PI3K/Akt/mTOR pathway [10]. These indicate that miR is involved in the development and progression of gastric cancer. miR-1228 is a tumor suppressor differentially expressed in breast cancer, liver cancer and other cancers [11–13]. The low expression of miR-1228 has been reported to be associated to the prognosis of patients with gastric cancer [14], but the specific mechanism has not been clarified.

Studies have proved the importance of microenvironment in gastric cancer [15]. Mesenchymal stem cells (MSCs) are adult non-hematopoietic stem cells capable of differentiating into bone, fat and cartilage tissue [16, 17]. They not only contribute to tissue repair and regeneration [18], but also are considered to be a key component in tumor microenvironment, which can participate in cancer progression through soluble factors generated during tumor occurrence [19]. According to Zhu et al., BM-MSCs are recruited to the tumor site and promote tumor growth in mouse xenograft models [20]. Exosomes are tiny membrane-bound extracellular vesicles that originate from multivesicular endosomes that fuse with invaginated plasma membrane to release them into extracellular matrix [21]. miRs in tumors can be secreted into blood, urine, breast milk, saliva, and other body fluids through exosomes [22]. Wang believes that serum exosomes are available biomarkers for gastric cancer [23]. Moreover, it has been reported that miRs in serum exosomes could be served as diagnostic markers for early gastric cancer [24].

Therefore, the clinical value of miR-1228 as well as the related mechanism of exosomal miR-1228 in gastric cancer were explored in order to provide references for clinical diagnosis and treatment.

## RESULTS

### Expression of miR-1228 in gastric cancer

**Figure 1:**
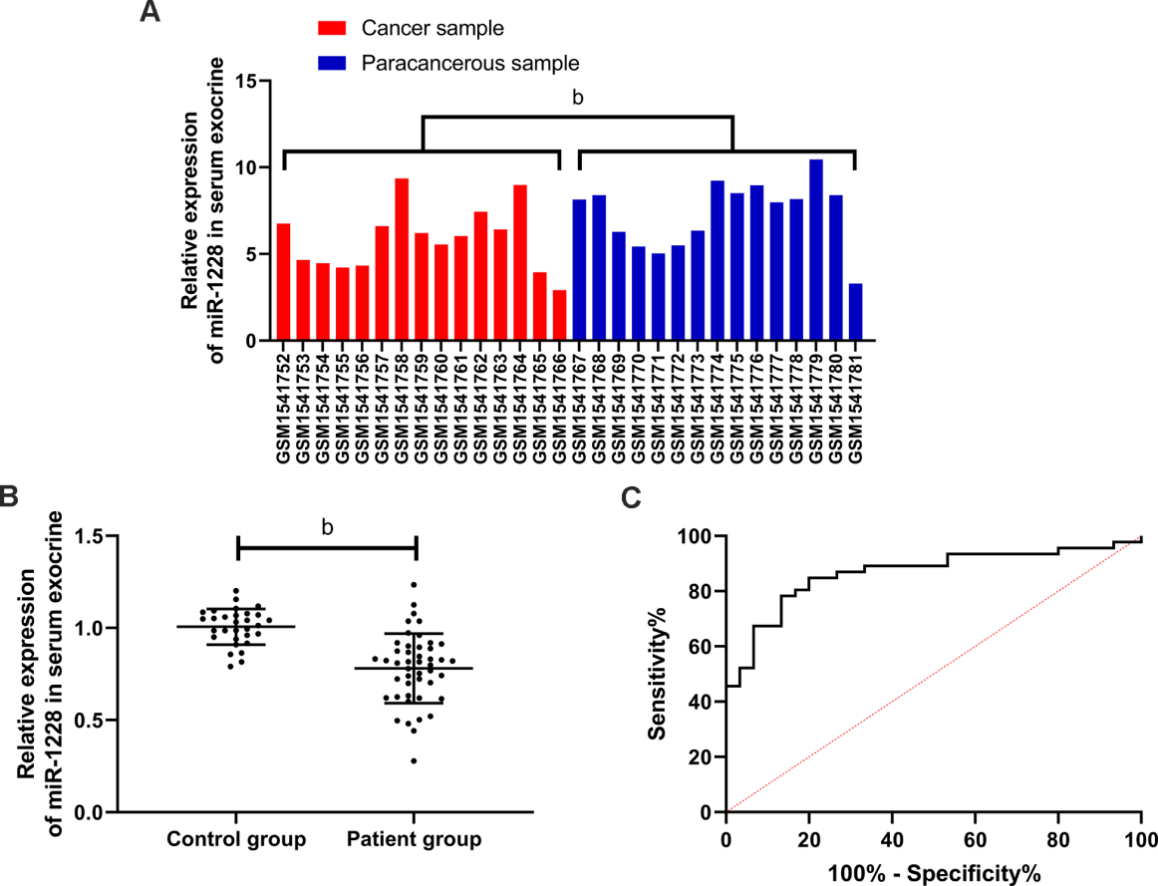
**Expression and diagnostic value of miR-1228 in gastric cancer.** (**A**) Expression of miR-1228 in microarray: red was cancer sample, blue was paracancerous sample. MiR-1228 was lowly expressed in cancer tissue. (**B**) Expression of miR-1228 in serum exosomes was significantly decreased in patients with gastric cancer. (**C**) Diagnostic value of miR-1228 in serum exosomes in gastric cancer. ^b^*P* < 0.01 between the two groups.

We found that miR-1228 was lowly expressed in gastric cancer via GEO database (Figure 1A). The expression of miR-1228 in serum exosomes decreased in the patient group (Figure 1B). According to the median value of the expression, the patients were divided into high and low expression groups to observe the relationship between the expression and the pathological data. In the low expression group, more patients were in stage III+IV, with lowly differentiated gastric cancer and increased probability of lymph metastasis (Table 1). ROC curves revealed that the AUC of miR-1228 in serum exosomes for diagnosing gastric cancer was 0.865 (Figure 1C).

**Table 1:** Correlation between expression of miR-1228 in serum exosomes and pathological data.

**Factor**		**Relative expression of miR-1228 in serum exocrine**		**c^2^ value**	**P value**
		**High expression group (n=23)**	**Low expression group (n=23)**		
Sex				1.533	0.216
	Male (n=30)	17(73.91)	13(56.52)		
	Female (n=16)	6(26.09)	10(43.48)		
Age				3.185	0.074
	≥60 years old (n=26)	10(43.48)	16(69.57)		
	< 60 years old (n=20)	13(56.52)	7(30.43)		
Tumor size				0.349	0.555
	≥3cm(n=22)	10(43.48)	12(52.17)		
	<3cm(n=24)	13(56.52)	11(47.83)		
Differentiation				7.263	0.007
	Moderately and highly differentiated (n=19)	14(60.87)	5(21.74)		
	Poorly differentiated (n=27)	9(38.13)	18(78.26)		
TNM staging				4.394	0.036
	I+II (n=19)	13(56.52)	6(26.09)		
	III+IV (n=27)	10(43.48)	17(73.91)		
Lymphatic metastasis				4.847	0.028
	Yes (n=15)	11(47.83)	4(17.39)		
	No (n=31)	12(52.17)	19(82.61)		

### Relationship between miR-1228 in serum exosomes and survival and prognosis

**Figure 2:**
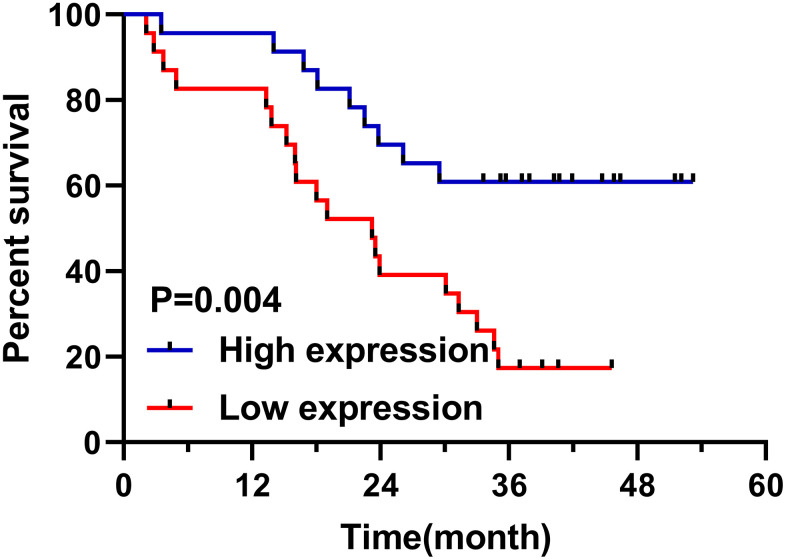
**Relationship between miR-1228 in serum exosomes and survival patients.** The overall survival in the low expression group was shortened.

Follow-up of 46 patients showed that 28 patients died, with a survival rate of 41.67% and an average survival time of 23.2 months. According to the median relative expression of miR-1228, patients were divided into high and low expression groups. And the low expression group was found to have a shorter overall survival (Figure 2). Univariate Cox regression showed that differentiation, TNM staging, lymphatic metastasis and miR-1228 were factors affecting prognosis (Table 2). While multivariate Cox regression confirmed that TNM staging and miR-1228 were independent risk factors affecting.

**Table 2:** Cox regression of prognostic factors of gastric cancer.

**Factor**	**Univariate Cox**			**Multivariate Cox**		
	**P value**	**HR**	**95CI%**	**P value**	**HR**	**95CI%**
Sex	0.962	0.982	0.459-2.100			
Age	0.101	1.950	0.879-4.325			
Tumor size	0.524	1.272	0.606-2.67			
Differentiation	0.029	0.400	0.175-0.911	0.376	0.661	0.265-1.652
TNM staging	0.008	0.329	0.144-0.753	0.035	0.402	0.173-0.936
Lymphatic metastasis	0.020	2.455	1.152-5.233	0.291	1.558	0.684-3.552
miR-1228	0.006	0.324	0.146-0.718	0.024	0.391	0.173-0.882

### Identification of human BM-MSCs

**Figure 3:**
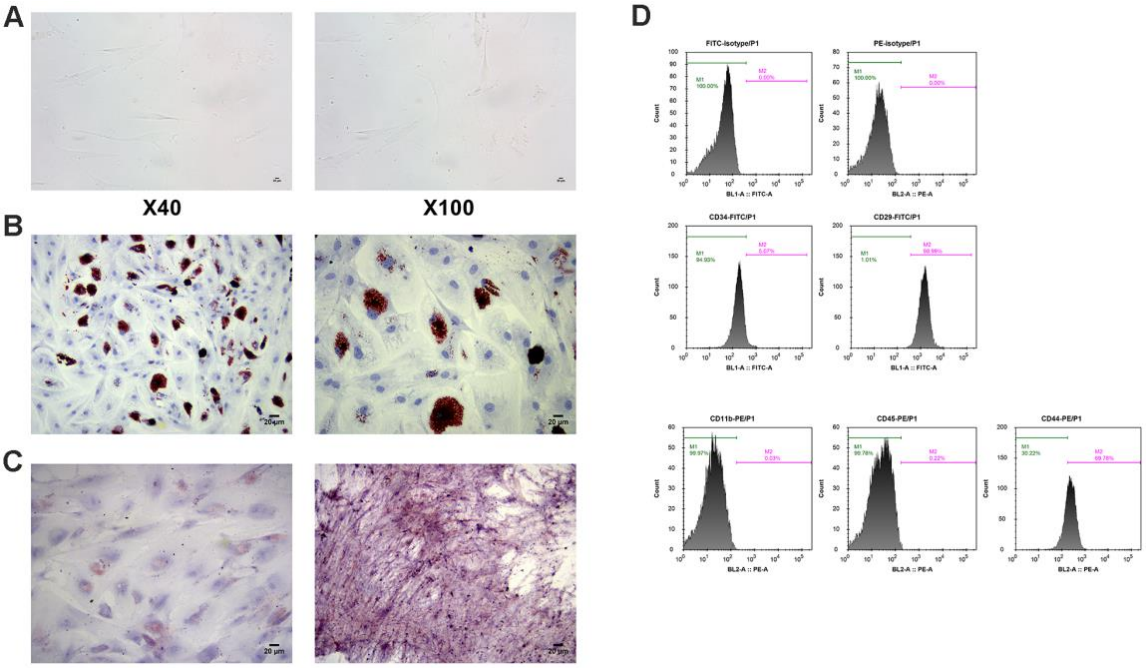
**Identification of MSCs.** (**A**) Morphology of MSCs under an optical microscope. (**B**) Identification of osteoblast differentiation of MSCs by alizarin staining. (**C**) Identification of adipogenic differentiation of MSCs by oil red O staining. (**D**) Measurement of cell-surface CD makers by flow cytometry. MSCs: mesenchymal stem cells.

The human BM-MSCs were fusiform and slender, and grew in whirlpool shape (Figure 3A). By examining cell-surface CD markers, it was found that the percentage of CD29 and CD44 positive was 98.99% and 69.78%, respectively. However, BM-MSCs were almost negative for hematopoietic markers CD34, CD11b and CD45 (Figure 3D). Alizarin red staining and oil red O staining identified the osteogenic and adipogenic differentiation abilities of MSCs (Figure 3B, 3C).

### Exosome identification

**Figure 4:**
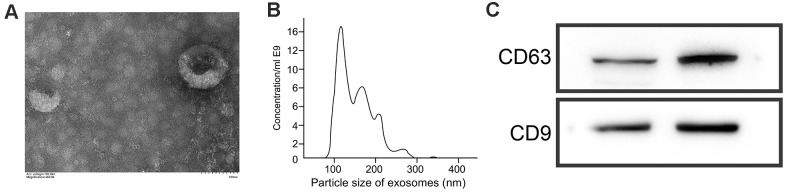
**Identification of exosomes.** (**A**) Morphology of exosomes under an electron microscope. (**B**) Analysis of particle size of exosomes by Brownian motion. (**C**) Quantification of CD63 and CD9 levels by WB.

CD9 and CD63 are important biological markers of exosomes. We detected their protein expression by WB, and found that both of them were expressed in exosomes (Figure 4C). Besides, we found that the diameter and distribution of exosomes were basically the same by Brownian motion, with a diameter of 20-120 nm (Figure 4A). Under a electron microscope, exosomes presented round or elliptic cell vesicles with a diameter of 50-100 nm (Figure 4B).

### Effects of miR-1228 in exosomes derived from human BM-MSCs on gastric cancer cells

**Figure 5:**
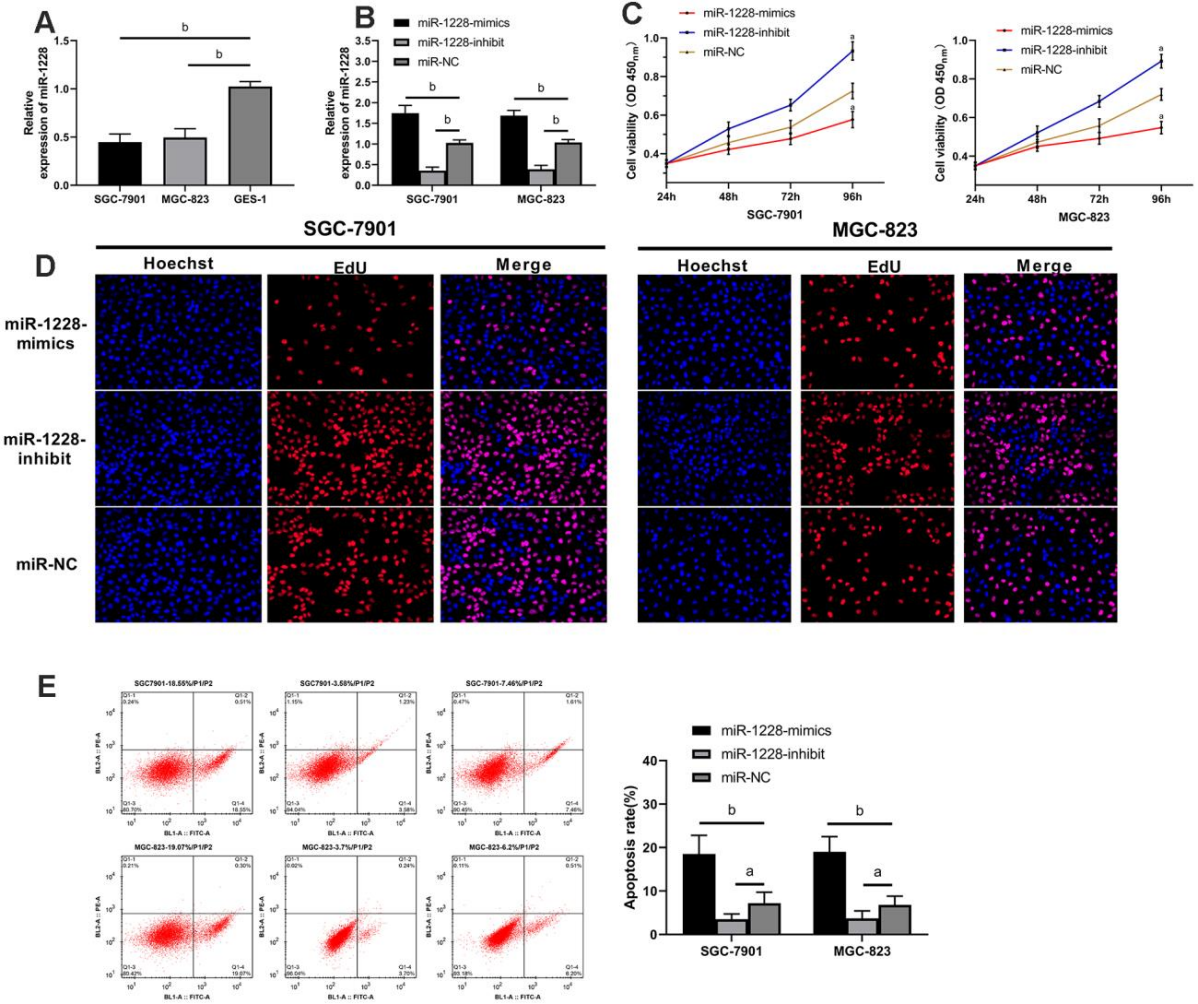
**Effect of exosomal-miR-1228 derived from BM-MSCs on growth and apoptosis of gastric cancer cells.** (**A**) miR-1228 was lowly expressed in gastric cancer cells. (**B**) Expression of miR-1228 in gastric cancer cells transfected with miR-1228-mimics, miR-1228-inhibit, miR-NC after co-culture with exosomes. (**C**) After co-culture, the cells transfected with miR-1228-mimics had a slower proliferation, while those transfected with miR-1228-inhibit had an accelerated proliferation. (**D**) As shown by EdU cell proliferation assay, the proliferation of cells transfected miR-1228-mimics was accelerated, while that of transfected miR-1228-inhibit was slowed down. (**E**) After co-culture, the cells transfected with miR-1228-mimics had an accelerated apoptosis, while those transfected with miR-1228-inhibit had a decreased apoptosis. ^a^*P*< 0.05 *vs.* miR-NC, ^b^*P*< 0.01 *vs.* miR-NC.

**Figure 6:**
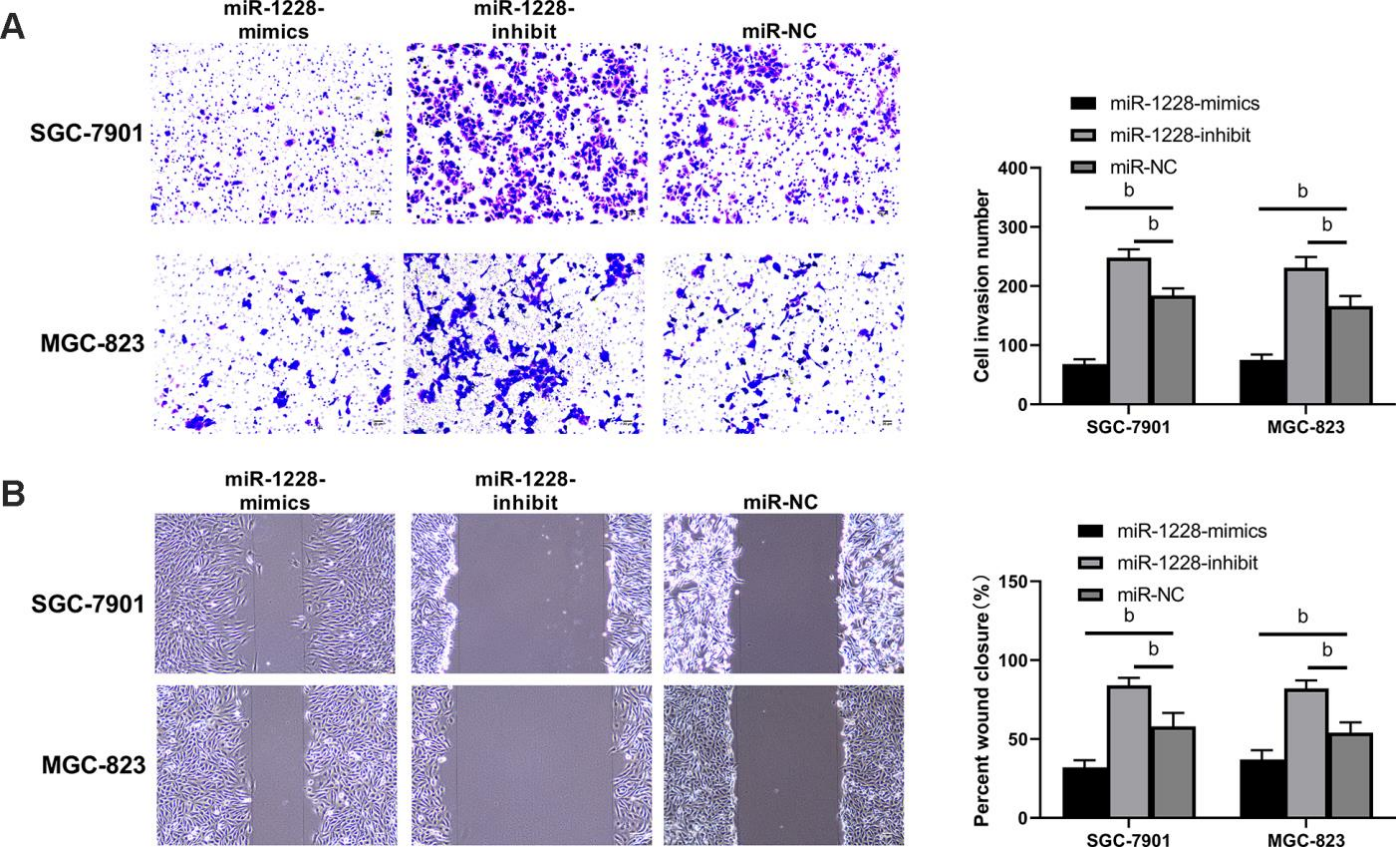
**Effect of exosomal-miR-1228 derived from BM-MSCs on invasion and migration of gastric cancer cells.** (**A**) After co-culture, the cells transfected with miR-1228-mimics had a decreased invasion, while those transfected with miR-1228-inhibit had an accelerated invasion. (**B**) After co-culture, the cells transfected with miR-1228-mimics had a decreased migration, while those transfected with miR-1228-inhibit had an accelerated migration. ^b^*P*< 0.01 *vs.* miR-NC.

The expression of miR-1228 in SGC-7901, MGC-823, and GES-1 cells was detected. SGC-7901 and MGC-823 cells showed remarkable lower miR-1228 expression than GES-1 cells (Figure 5A). After co-culture with exosomes, SGC-7901 and MGC-823 cells transfected with miR-1228-mimics had a significantly higher miR-1228 expression than those transfected with miR-NC, while the expression in cells transfected with miR-1228-inhibit was lower than those transfected with miR-NC (Figure 5B). Further detection of cell biological functions found that after co-culture the proliferation (Figure 5C, 5D), invasion (Figure 6A) and migration (Figure 6B) of cells transfected with miR-1228-mimics were significantly inhibited and the apoptosis (Figure 5E) was significantly increased, while opposite results were obtained in cells transfected with miR-1228-inhibit.

### Exosomal miR-1228 derived from human BM-MSCs targets MMP-14 to regulate the growth of gastric cancer cells

**Figure 7:**
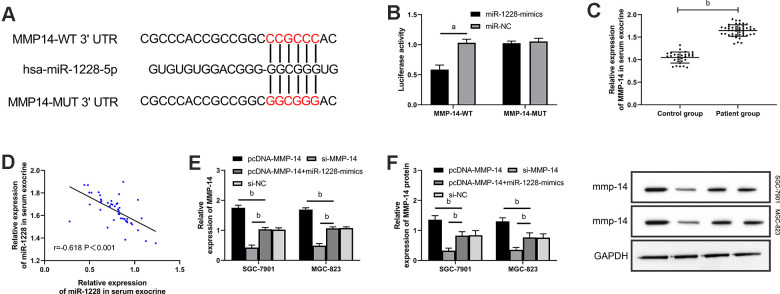
**miR-1228 targets and regulates MMP-14. ** (**A**) Dual-luciferase reporter assay confirmed that there were targeted binding sites between miR-1228 and MMP-14. (**B**) Relative expression of MMP-14 mRNA in gastric cancer cells (transfected with sh-MMP-14, si-MMP-14, si-NC, or sh-MMP-14+miR-1228-mimics) after co-culture with exosomes. (**C**) qRT-PCR quantitated relative expression of MMP-14in serum exosomes. (**D**) Pearson test analyzed the correlation between MMP-14 and miR-1228 in serum exosomes. (**E**, **F**) qRT-PCR and WB determined relative expression of MMP-14 mRNA and protein in gastric cancer cells (transfection with sh-MMP-14, si-MMP-14, si-NC or sh-MMP-14, miR-1228- mimics) after co-culture. ^a^*P*< 0.05 *vs.* si-NC, ^b^*P*< 0.01 *vs.* si-NC.

**Figure 8:**
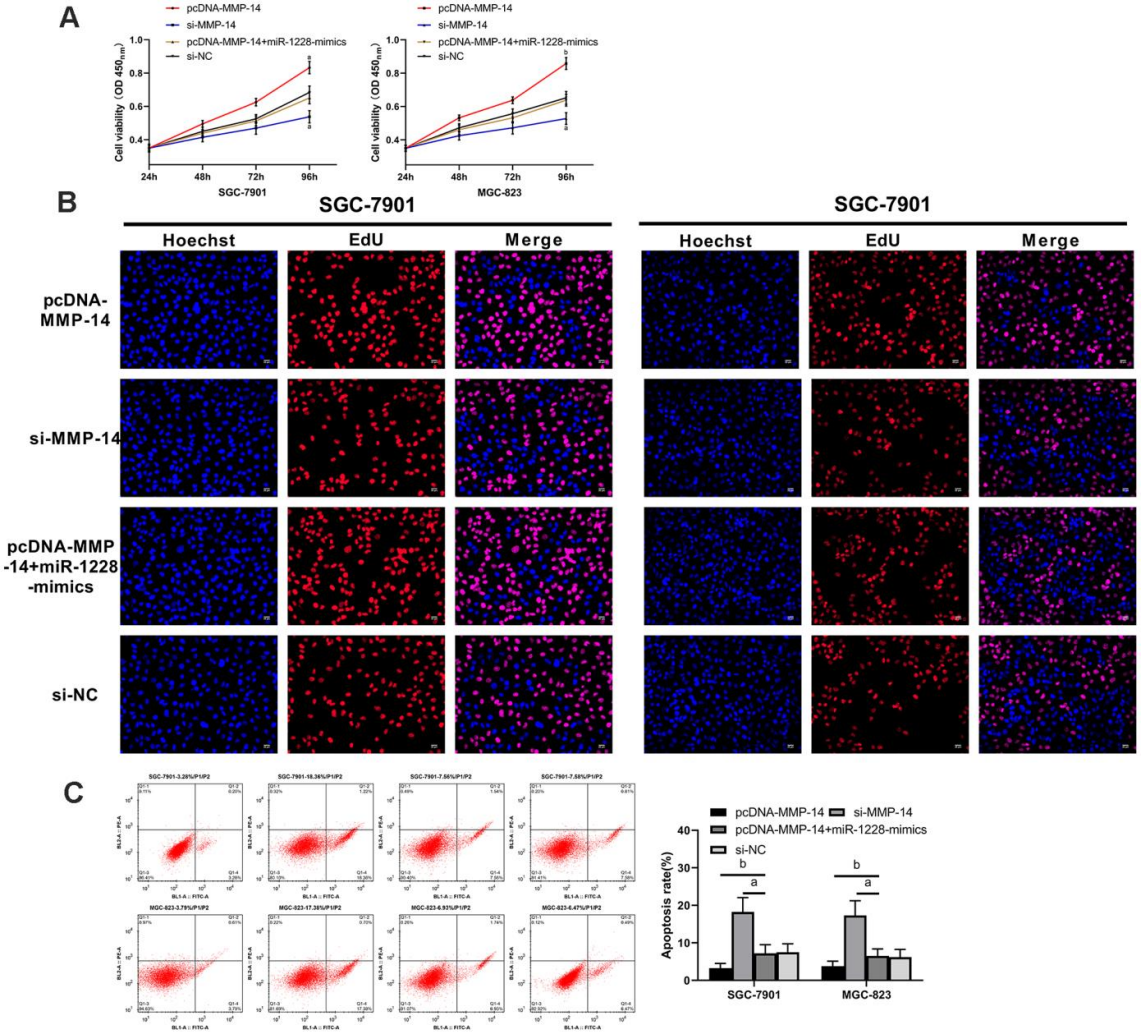
**Overexpression of miR-1228 inhibits MMP-14 upregulation-induced growth and apoptosis of gastric cancer cells.** (**A**, **B**) The proliferation of si-MMP-14 transfected cells was slowed down after co-culture, while that of sh-MMP-14 transfected cells was accelerated. (**C**) After co-culture, the apoptosis of si-MMP-14 transfected cells was accelerated and that of sh-MMP-14 transfected cells was slowed down. The apoptosis of sh-MMP-14+miR-1228-mimics transfected cells had no difference from that of si-NC transfected cells. ^a^*P*< 0.05 *vs.* si-NC, ^b^*P*< 0.01 *vs.* si-NC.

**Figure 9:**
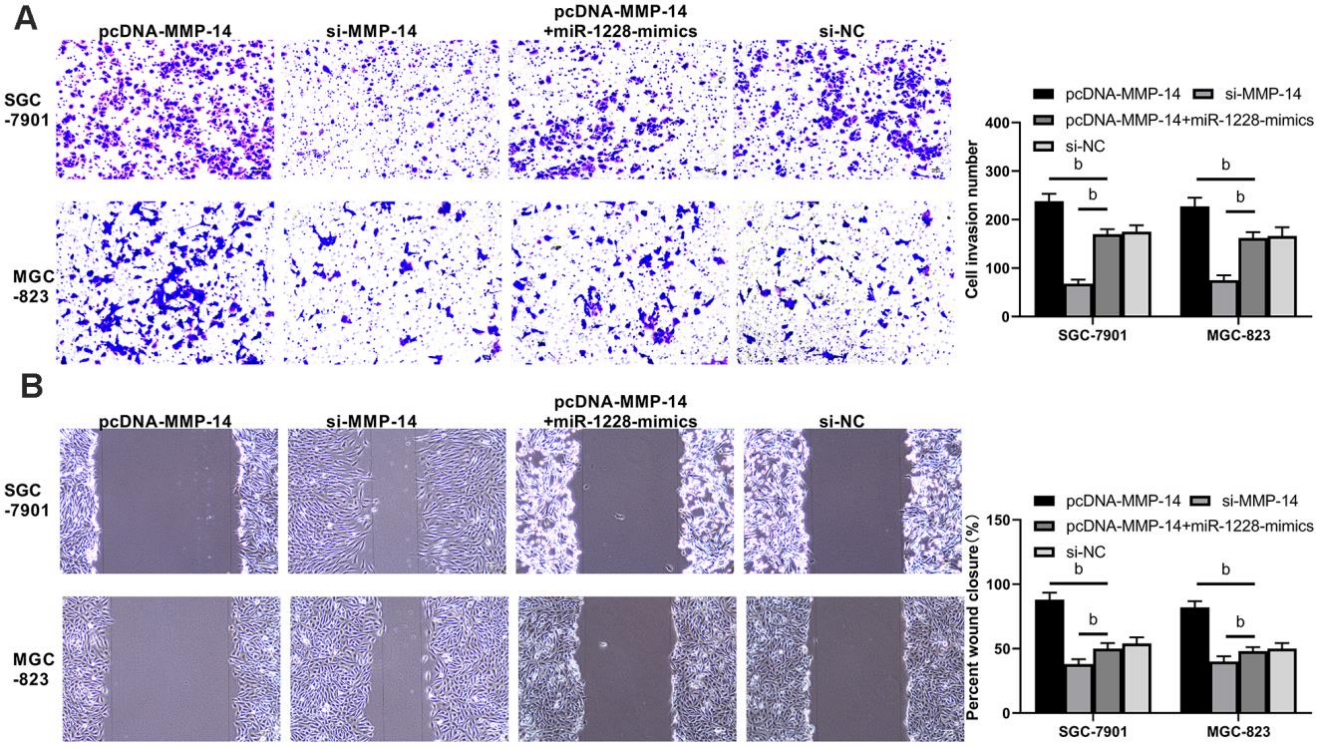
**Overexpression of miR-1228 inhibits MMP-14 upregulation-induced invasion and migration of gastric cancer cells.** (**A**) The invasion of si-MMP-14 transfected cells was slowed down after co-culture, while that of sh-MMP-14 transfected cells was accelerated. The invasion of sh-MMP-14+miR-1228-mimics transfected cells had no difference from that of si-NC transfected cells. (**B**) After co-culture, the migration of si-MMP-14 transfected cells decreased while that of sh-MMP-14 transfected cells increased. ^b^*P*< 0.01 *vs.* si-NC.

We found that there were targeted binding sites between miR-1228 and MMP-14 with an on-line target gene prediction tool (Figure 7A). Dual-luciferase reporter assay demonstrated that the fluorescence activity of MMP-14-WT was significantly reduced (Figure 7B). qRT-PCR indicated increased MMP-14 level in serum exocrines. And correlation analyses showed that MMP-14 and miR-1228 were negatively correlated in serum exocrines of patients with gastric cancer (Figure 7C, 7D). Cells transfected with sh-MMP-14 had a significantly increased MMP-14 expression after co-culture with exosomes, while that in cells transfected with si-MMP-14 was decreased (Figure 7E, 7F). Observing the effect of MMP-14 on cell growth, it was found that the proliferation (Figure 8A, 8B), invasion (Figure 9A), and migration (Figure 9B) of cells transfected with sh-MMP-14 and co-cultured with exosomes were significantly accelerated and the apoptosis (Figure 8C) was reduced, while the cells transfected with si-MMP-14 got the reversed result. To verify the result that miR-1228 affects the growth of gastric cancer cells via regulating MMP-14, we cultured exosomes along with miR-1228-mimics+sh-MMP-14 transfected gastric cancer cells. The results showed that miR-1228-mimics suppressed the promotion of sh-MMP-14 in cell proliferation, invasion, and migration, and accelerated apoptosis, with no difference from si-NC.

## DISCUSSION

In the present study, miR-1228 was found to be lowly expressed in patients with gastric cancer via GEO database, and clinical trial on detection of the expression of miR-1228 in serum exosomes obtained the same result. Jia et al. reveal that miR-1228 has a low expression in gastric cancer and weakens the pro-angiogenic activity of gastric cancer cells by targeting macrophage migration inhibitory factors [13], indicating that miR-1228 participates in the progression of gastric cancer. However, the relationship between miR-1228 in serum exosomes and gastric cancer has not been reported. To determine the value of miR-1228 in serum exosomes, we divided the patients into high and low expression groups by its median expression. In the low expression group, more patients were in stage III+IV, with lowly differentiated gastric cancer and increased probability of lymph metastasis. Moreover, the AUC was more than 0.9, and the overall survival of patients with low expression was shortened. These suggest that serum exosomal miR-1228 is a potential diagnostic and prognostic marker for gastric cancer. According to the above results, the clinical value of serum exosomal miR-1228 in gastric cancer was confirmed, but the mechanism was not.

MSCs, especially human BM-MSCs, are an essential component of tumor microenvironment, which play an vital role in maintaining cancer stem cells and tumor growth, angiogenesis, and metastasis [25]. In the research of Figueroa et al., exosomes derived from glioma-associated mesenchymal stem cells increase tumorigenicity of glioma stem cell-like cells by transferring miR-1587 [26]. Besides, Lv et al. point out that miR-21 promotes migration of BM-MSCs via activating PI3K/Akt/MMPs pathway [27]. Therefore, understanding the biological characteristics of MSCs is helpful to the development of effective cancer treatments. As extracellular carriers, exosomes can mediate long-distance cellular communication without direct contact with cells [28]. In addition, they are considered as natural nanocarriers with biocompatibility [29]. miRs are a class of short non-coding RNAs that regulate tumor growth by inhibiting transcription of downstream target genes [30]. A variety of carcinogenic miRs are widely present in exosomes, inducing the growth and metastasis of inflammatory hormone tumors. According to Yu et al., exosomes derived from miR-199a-overexpressing MSCs inhibit glioma progression through down-regulating AGAP2 [31], and Lou et al. state that exosomes derived from miR-122-modified adipose tissue-derived MSCs increase chemosensitivity of liver cancer [32]. These findings provide research directions for diagnosis and treatment of exosomes-based malignant tumors by miRs.

In the present study, exosomes were extracted from transfected human BM-MSCs and co-cultured with gastric cancer cells. It turned out over-expression of miR-1228 inhibited proliferation, invasion, migration and accelerated apoptosis of gastric cancer cells. Furthermore, we found that MMP-14 was a potential downstream target of miR-1228 through Bioinformatics Online. MMP-14 is an important member of matrix metalloproteinases family. Kasurinen et al. indicate the association between high expression of MMP-14and poor prognosis of patients with gastric cancer [33], while Zheng et al. mention that miR-584-3p inhibits the progression of gastric cancer by inhibiting Yin Yang 1-facilitated MMP-14 expression [34]. In this study, over-expression of miR-1228 inhibited the expression of MMP-14 protein in gastric cancer cells, while suppression of miR-1228 expression increased it, which suggests that miR-1228 participates in the progression of gastric cancer by regulating MMP-14. In order to verify the role of miR-1228 in regulating MMP-14, we co-cultured exosomes derived from miR-1228-mimics and sh-MMP-14 transfected human BM-MSCs with gastric cancer cells. The results showed that after co-culture, the effects of sh-MMP-14 on promoting proliferation, invasion, and migration of gastric cancer cells were weakened, thus accelerating apoptosis. Therefore, the over-expression of miR-1228 inhibited the growth of gastric cancer cells by down-regulating the expression of MMP-14.

However, there are still several limitations in this study. Firstly, nude mouse tumorigenicity assay is not conducted, so the effect of over-expression of miR-1228 in human BM-MSCs on solid tumors remains unknown. Secondly, patients with benign gastric lesions are not included, and the expression of serum exosomal miR-1228 in those patients is still unclear. Finally, the sample size in this study is small, so more samples need to be collected to confirm our findings. Therefore, we hope to increase corresponding basic experiments and enroll more kinds and quantities of samples to validate our results.

## CONCLUSIONS

In conclusion, exosomes derived from human BM-MSCs can be used as efficient nanocarriers, and serum exosomes are potential nanocarriers for regulating the *in vivo* expression of miR-1228. Moreover, the controlled expression of miR-1228 in serum exosomes by inhibiting MMP-14 is expected to be an available treatment for gastric cancer.

## MATERIALS AND METHODS

### Collection of microarray data from GEO database

Login to https://www.ncbi.nlm.nih.gov/gds to download miR gene chip (GSE63121) of gastric cancer, and merge it into matrix file. Input hsa-miR-1228 and click “Search”, then analyze the data extracted.

### Collection of clinical data

Forty-six patients with gastric cancer who were treated in Shijiazhuang First Hospital from March 2014 to June 2015 were enrolled as the patient group, and another 30 healthy individuals who underwent physical examinations during the same period were enrolled as the control group. The patient group consisted of 30 males and 16 females (average age 61.7±7.2 years), whereas the control group consisted of 20 males and 10 females (average age 60.4±6.2 years). This study was conducted with the approval of the Medical Ethics Committee of Shijiazhuang First Hospital. Inclusion criteria: patients diagnosed with gastric cancer through pathological and imaging examinations and undergoing no radiotherapy and chemotherapy before this study, as well as those who were informed of this study and signed the informed consent form. Exclusion criteria: patients with other tumors, congenital defects or chronic diseases, as well as those who did not cooperate with follow-up or with a expected survival of shorter than 1 month. The patients were followed up by telephone and outpatient review in the 1st, 3rd, 6th, 9th and 12th months of each year until January 2019.

### Cell culture

Gastric cancer cell lines (SGC-7901, MGC-823) and normal cell line (GES-1) (ATCC, USA) were grown in Dulbecco’ s Modified Eagle Medium (DMEM; Thermo Fisher Scientific) containing 10% fetal bovine serum (FBS; Gibco, USA) and cultured in a 37° C and 5% CO_2_ incubator.

### Collection and identification of human BM-MSCs

BM-MSCs from healthy subjects (patients know and sign the informed consent form) were collected and diluted with an equal volume of phosphate buffer saline (PBS). Then the cells were separated with 1.077g/mL Ficoll (Sigma, USA) and centrifuged at 2000rpm for 20 min, and the supernatant was discarded. After a PBS rinse, the collected cells were placed in DMEM (Cyagen Biosciences, Guangzhou, China) containing 10% FBS and cultured in a 37° C and 5% CO_2_ incubator. The cells in passage 4 were harvested for following experiments (the medium was changed on the 3rd day of primary cell culture and once every 2 days thereafter). Flow cytometry was applied to identify specific cell-surface markers CD29, CD44, CD73, CD90 and CDl05. The collected cells were resuspended to a density of 5*10^4^ and then inoculated in a 12-well plate. After reaching 100% confluence, the cells were respectively inoculated to adipocytes and osteocytes for induction culture, and the medium was changed once every 3 days. The differentiation was identified with oil red O and alizarin red staining on the 9th and 21st days of culture.

### Cell transfection

Lentiviral vectors carrying miR-1228-mimics (over-expression sequence), miR-1228-inhibit (inhibition sequence), miR-NC (negative control), si-MMP-14 (inhibition sequence), MMP-14 overexpression (pcDNA-MMP-14), si-NC (negative control) were constructed. Primer sequences were designed and synthesized by Shanghai Sangon Biology Co., Ltd. Construction methods: The HEK-293T cells were co-transfected with lentiviral vectors pRSV-Rev, pMD2.G, or pMDLg/pRRE plasmids (Biofeng, CHN) to obtain lentiviral particles. The human BM-MSCs cells in passage 4 were inoculated in a 24-well plate and cultured for 24 hours, then added with lentivirus suspension (multiplicity of infection [MOI] = 20) and polyamine (5μg/ mL). Cells were cultured for additional 24 h, then the medium containing lentiviral particles was replaced with fresh medium. The transfection efficiency was estimated under a fluorescence microscope (Thermo Fisher Scientific, USA).

### Extraction and identification of exosomes

After transfection, human BM-MSCs cells and peripheral blood (5mL) of patients were centrifuged at 2000rpm and 4° C for 10 min to obtain supernatant. Afterwards, ExoQuick-TC™Exosome precipitation solution (system Biosciences, USA) was employed to extract exosomes. The specific operations were as follows: the collected supernatant was centrifuged at 11000 rpm for 30 min, and cell debris was removed. EXOTC10A-1(System Biosciences) reagent and the supernatant (1:5) were mixed evenly and stored at 4° C for at least 12 h. The mixture was centrifuged at 3000 rpm for 30 min, then the exosomes were collected at the tube bottom then stored in PBS (-80° C). Proteins in exosomes were extracted with RIPA lysis buffer (Thermo Fisher Scientific), and the protein expression of CD9 and CD63 were detected by Western blot (WB). The concentration and size of the exosomes were determined by Brownian motion on a Microtrac S3500 analyzer (Microtrac, Montgomeryville, PA, USA).

### Co-culture of exosomes with SGC-7901 and MGC-823 cells

Gastric cancer cell lines SGC-7901 and MGC-823 were transfected with fluorescently labeled pCDNA3.1-GFP, and human MSCs were transfected with Cy3-labeled miR-1228 (miR-1228-Cy3) (GenePharma, Shanghai, China). Twelve hours later, all cells were mixed at 1:1 and inoculated into a 96-well plate (100cells / well) for 2 days. The cells were sorted by flow cytometry and analyzed under a fluorescence microscope. At last, 2 μg of exosomes were co-cultured with SGC-7901 and MGC-823 cells for 48 h.

### Cell proliferation (CCK-8)

The Cell counting kit-8 (CCK-8) (Beyotime Biotechnology, Shanghai, China) was applied to determine cell proliferation. Cells cultured for 24 hours were collected and planted on a 96-well plate (4*10^6^ cells/well). CCK8 solution (10 μL) and basic medium (DMEM, 90μL) were added to each well after 24, 48, 72, and 96 hours of inoculation, The cells were cultured at 37° C for 2 h. Afterwards, optical density (OD) values were read at 450nm using a microplate reader.

### EdU cell proliferation assay

To measure the proliferation of cells, we used Cell-Light EdU imaging kit (RiboBio, Guangzhou, China) to test EdU incorporation. Steps were briefly outlined as follows: after 24 h of transfection, MGC-823 and SGC-7901 cells were selected from the co-culture system and washed twice with PBS, and then seeded into a 96-well plate (5 × 10^3^ cells / well). After 6 h, the medium containing EdU was added to the plate and incubated for 60 min. Next, the cells were fixed with 4% formaldehyde for 15 min, treated with 0.5%Triton X -100 for 20 min, and stained with Apollo solution for 30 min. In each well, DNA was stained with 5 μp/mL Hoechst 33342 for 30 min. Images were captured and analyzed under a fluorescence microscope (excitation at 350 nm and emission at 461 nm).

### Cell invasion and migration

The Transwell kit (Gibco Company, USA) was used for detection of cell invasion. Cells cultured for 24 h were planted on a 6-well plate (5*10^4^ cells/well), washed twice with PBS, and inoculated in the upper chamber. The upper chamber was added with 200 μL of DMEM, while the lower chamber was added with 500 mL of DMEM (containing 20%FBS). After incubation at 37 ° C for 48 h, the matrix and cells not penetrating the membrane in the upper chamber were wiped off. The remaining cells were rinsed 3 times with PBS, fixed with paraformaldehyde for 10 min, rinsed another 3 times with double distilled water, then air dried and stained with 0.5% crystal violet. A microscope was employed to observe cell invasion. Cells cultured for 24 h were planted on a 6-well plate (2x10^5^ cells/well). After 24 h, a pipette tip was used to make a straight line along the diameter of the plate. Floating cells were removed lightly with PBS. Under a 20x microscope, 5 fields of view were randomly selected in each well for imaging. Then the cells were cultured in serum-free medium for 36 h, and the imaging was carried out in the same way as mentioned above.

### Cell apoptosis

Cells cultured for 24 h were digested with 0.25% trypsin, washed twice with PBS, and added with 100 μL of binding buffer to prepare suspension (1*10^6^ cells/mL). After addition of AnnexinV-FITC (Yeasen Biotechnology Co., Ltd., Shanghai, China) and PI in turn, the suspension was incubated at room temperature for 5 min in the dark. Cell apoptosis was detected by a FC500MCL flow cytometer. The test was repeatedly conducted 3 times to obtain the average.

### RT-qPCR

Total RNAs were extracted from the collected cells and serum exosomes using TRIzol reagent (Invitrogen, USA). The concentration, integrity, and purity were measured by agarose gel electrophoresis and an UV spectrophotometer. Reverse transcription (Invitrogen, USA) was performed with a TaqMan Reverse Transcription Reagents kit, and SYBR _ PREMIX EXTAQ II (Takara, Dali, China) and ABI 7500PCR (Applied Biosystems, USA) were adopted for amplification. Amplification system: 10 μL of SYBR Premix Ex Taq II (2X), 2 μL of cDNA, 0.8 μL each of upstream primer and downstream primer, and supplemented to 20 μL with sterile purified water. Amplification conditions: pre-denaturation at 95° C for 30 s, denaturation at 95° C for 5 s, annealing and extension at 60° C for 30 s, for 40 cycles. Three replicate wells were set up for each sample and the experiment was repeatedly conducted 3 times. U6 was used as miR internal reference, and 2^-ΔΔCT^ was used for data analysis [35].

### Western blot (WB)

The cultured cells were harvested to extract total proteins with RIPA lysis (Thermo Scientific, USA, and their concentration was detected using bicinchoninic acid method (Thermo Scientific, USA), then adjusted to 4μg/μL. Following separating with 12% SDS-PAGE, the proteins were transferred to a polyvinylidene difluoride membrane. Subsequently, the membrane was dyed in Ponceau S working solution, soaked in PBST for 5 min and washed, sealed with 5% non-fat milk powder for 2 h, and incubated overnight at 4° C along with primary antibodies against CD9 and CD63, MMP-14 (1: 1000, ABCM, USA). After washing the membrane to remove the primary antibodies, horseradish peroxidase labeled goat anti-rabbit (Abcam, USA) secondary antibody (1: 5000) was added for another 1-h incubation (37° C). Afterwards, the membrane was washed 3 times with PBS for 5 min each, and the excess liquid was dried with a filter paper. The development was performed with enhanced chemiluminescence (ECL) in a darkroom. Gray values of luminescent protein bands were measured with Quantity One. Relative expression of target proteins = gray value of target protein band /gray value of β-Actin protein band.

### Dual-luciferase reporter assay

MMP-14 was cloned into pmirGLO dual-luciferase target expression vector by a Lipofectamine™ 2000 kit (Invitrogen, USA). PmirGLO-MMP-14-3'UTR wild type (Wt) and pmirGLO-MMP-14-3'UTR mutant (Mut) were respectively constructed and transferred to the downstream of luciferase reporter genes to sequence the constructed plasmids. MGC-823 cells were transfected with correctly sequenced plasmids together with miR-1228-mimics or miR-NC. A dual-luciferase reporter gene assay kit was employed to determine the luciferase activity (Solarbio, Beijing, China).

### Statistical analysis

Statistical analysis was performed using SPSS20.0 software package, and GraphPad 7 software package was used to draw figures. The measurement data distribution was analyzed by Kolmogorov-Smirnov (K-S) test. Normally distributed data were expressed by means±standard deviation (Mean±SD), and the comparison between groups was conducted with independent samples t test. While non-normally distributed data were expressed by quartile [M (P25~P75)] and analyzed with non-parametric test (denoted by Z). The multi-group comparison was conducted with one-way analysis of variance (ANOVA) (denoted by F), and post-hoc pairwise comparison was conducted with Fisher’s least significant difference-t test. Expression at multiple time points was analyzed with repeated measures ANOVA (denoted by F), and the post hoc test was carried out with Bonferroni. Receiver operating characteristic (ROC) curves were applied to assess the diagnostic value of miR-1228 in serum exocrines of patients with gastric cancer. Pearson test was used to analyze the correlation between miR-1228 and MMP-14, and Kaplan-Meier survival curve and Log-rank test to evaluate the survival rate, and multivariate Cox regression to analyze the prognosis of patients. A value of *P* < 0.05 indicated a statistical difference.
